# Efficacy of adalimumab for Hailey-Hailey disease demonstrated by reduction of the Pemphigus Disease Area Index

**DOI:** 10.1016/j.jdcr.2024.11.025

**Published:** 2024-11-30

**Authors:** Madeleine Stark, Tabrez Sheriff, Dédée F. Murrell

**Affiliations:** a Department of Dermatology, St George Hospital, Sydney, New South Wales, Australia; b Faculty of Medicine, University of New South Wales (UNSW), Sydney, New South Wales, Australia

**Keywords:** adalimumab, bullous disease, genodermatoses, Hailey-Hailey disease, Pemphigus Disease Area Index (PDAI)

Hailey-Hailey disease (HHD), or benign familial chronic pemphigus, is a rare autosomal dominant blistering genodermatosis described in 1939 by the Hailey brothers. It is caused by haploinsufficiency of the enzyme ATP2C1 on chromosome 3 that encodes the human secretory pathway Ca^2+^/Mn^2+^-ATPase protein 1. This leads to impaired intracellular calcium regulation and subsequent malformation of intercellular desmosomes.^1^ This defect results in acantholysis and blistering that commonly develops in areas of increased friction such as the skinfolds. To date, no validated severity score has been developed or tested in HHD. The lesions are somewhat similar to pemphigus foliaceus (PF), albeit that the distribution of lesions is more flexural and not on the face. Only a Physician Global Assessment score without definitions has been reported.^2^

As there is no definitive cure, treatment is generally aimed at minimization of exacerbating factors, inflammation and mitigation of symptoms and secondary infections. Alongside lifestyle advice, treatment modalities can be divided into topical, local, and systemic. Topical options include antiseptic washes and topical corticosteroids and calcineurin inhibitors as well as UV light therapy; local options include intralesional corticosteroids, laser therapy, dermabrasion, botulinum A toxin and, in severe cases, surgery. Systemic options include anti-inflammatory antibiotics, methotrexate, dapsone, cyclosporin, oral retinoids, glycopyrrolate, low-dose naltrexone, and magnesium chloride.^1^ Increases in the proinflammatory cytokines tumor necrosis factor-alfa (TNF-α) and interleukin 6 (IL-6) have been suggested in the pathophysiology of HHD.^3^^,^^4^ Various cytokine inhibitors and modulators have been trialed and reported to improve HHD, including apremilast, dupilumab, etanercept, and JAK inhibitors.^2^^,^^5^^,^^6^

We report a case of a 55-year-old woman with HHD who has been symptomatic since her 20s, with multiple family members affected by HHD. She presented to our Dermatology department with erythema and erosions, with crusting over her back, submammary region and axillae. Biopsies from 2 sites demonstrated acantholytic dyskeratosis, consistent with HHD.

She experienced severe flares requiring multiple courses of oral prednisolone, developed multiple secondary bacterial and viral infections, and significant cutaneous pain requiring management by pain specialists.

She was trialed on numerous treatments including dapsone, methotrexate, acitretin, low-dose naltrexone, and UV-B therapy. Methotrexate and acitretin had been effective but were discontinued because of liver function derangement. Naltrexone was ceased when her pain requirement escalated to requiring opioid medications. She continued on glycopyrrolate 1 mg once daily, doxycycline 100 mg twice daily, valaciclovir 500 mg twice daily alongside topical corticosteroids.

Her other regular medications include dabigatran 150 mg twice daily, due to a history of recurrent venous thromboembolism. She also unfortunately experienced many glucocorticoid toxicities secondary to prolonged oral corticosteroid use. As a result, she was commenced on denosumab and calcium/vitamin D supplementation to manage osteoporosis, ramipril 2.5 mg once daily for hypertension, rosuvastatin for hypercholesterolaemia and pantoprazole 40 mg once daily for gastro-esophageal reflux disease.

A recent case report published by Chen et al^7^ demonstrated marked improvement in HHD when commenced on adalimumab for management of ulcerative colitis. Thus, because of the severe and refractory nature of her condition we obtained permission from the Therapeutic Goods Association of Australia to trial adalimumab, an anti–TNF-α agent, at 40 mg every 2 weeks subcutaneously.

The Pemphigus Disease Area Index (PDAI) scoring tool was used to assess efficacy of adalimumab in our patient. The PDAI was developed by the International Pemphigus Definitions Committee over a 3-year period in 2008.^8^ It consists of scoring disease *activity* (new erythema, erosions or blisters) and *damage* (postinflammatory hyperpigmentation or erythema) in the skin and scalp. Mucus membranes are also scored for activity only. The scoring system has been validated for use in autoimmune blistering diseases and is being used in clinical practice to guide treatment and in clinical trials to assess the efficacy of new drugs for pemphigus. Similar to PF, HHD presents with erythema, erosions, blisters, and crusting with lesions healing with postinflammatory pigmentation. However, the distribution of HHD differs from that of PF and pemphigus vulgaris, affecting the intertriginous zones, and generally sparing the face and mucous membranes. Development of a specific assessment tool for blistering genodermatoses would be beneficial to more accurately assess HHD activity. However, due to the dearth of options specifically developed for the assessment of HHD and the similarities in presentation between PF and HHD, we decided to use the PDAI score in this case to provide a more objective measure of disease extent and activity.

After approximately 12 weeks of 40 mg of adalimumab every 2 weeks, our patient reported significant symptomatic improvement which is illustrated in Figures 1 and 2 below. There was also an improvement in her PDAI activity and damage scores, as demonstrated in Figure 3.

**Fig 1:**
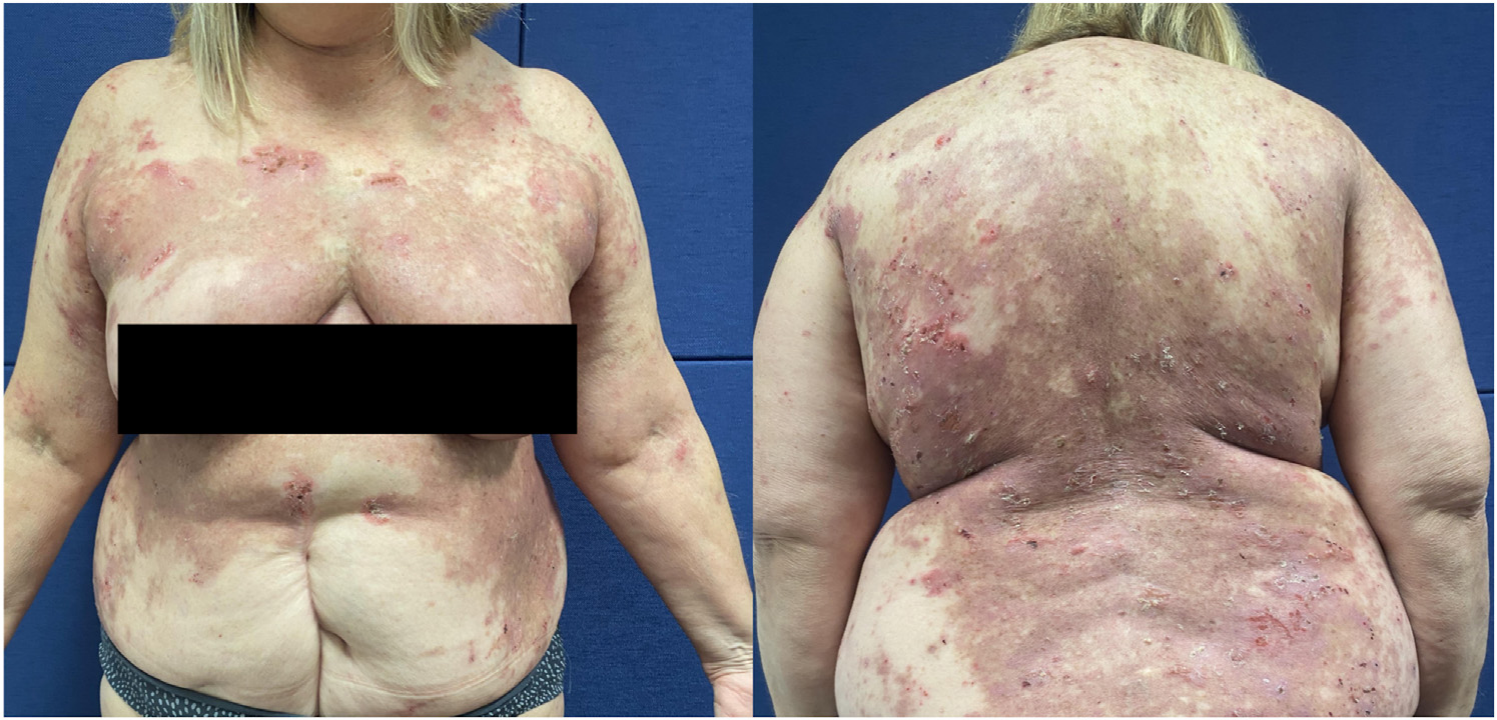
Prior to commencement of adalimumab. Extensive erythema and erosions over torso. PDAI: Activity 22, Damage 8. *PDAI*, Pemphigus Disease Area Index.

**Fig 2:**
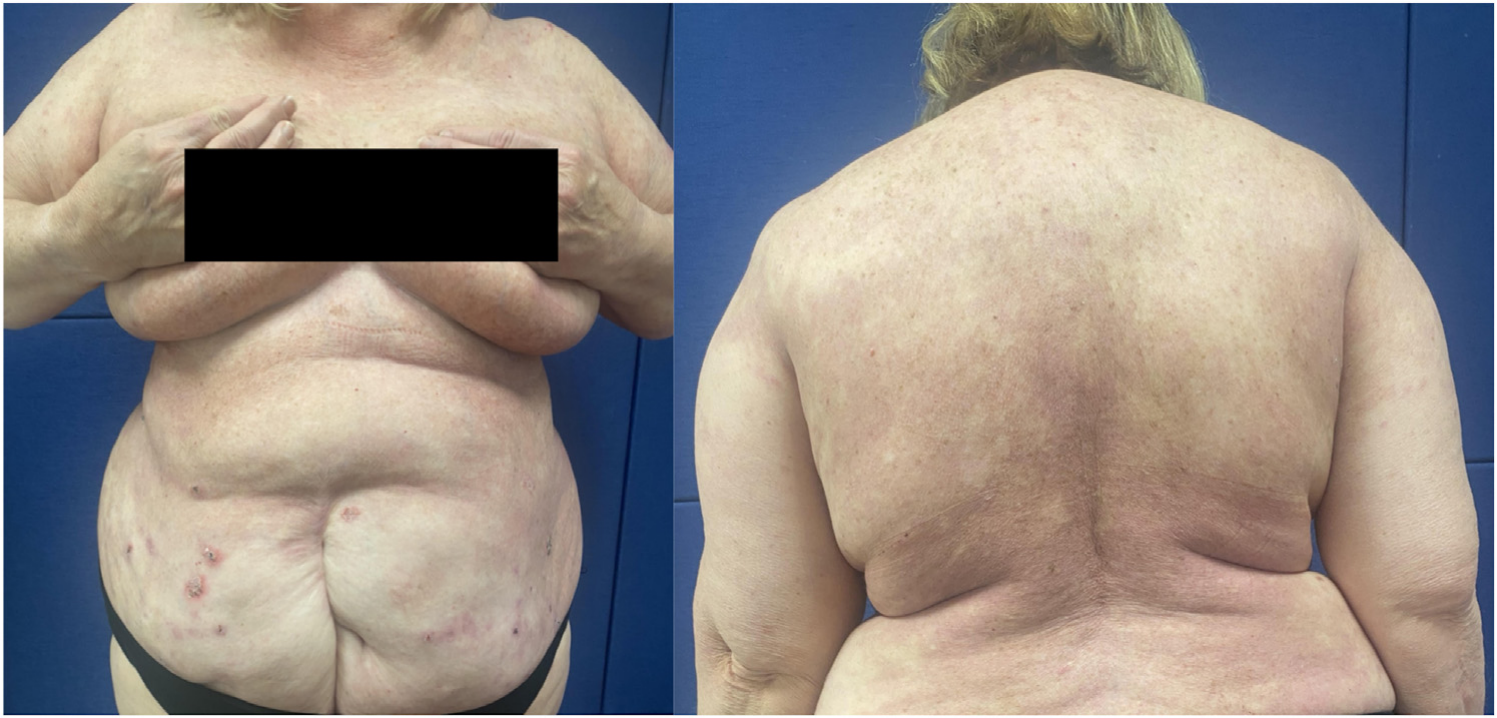
Significant improvement after 12 weeks of treatment with adalimumab. Postinflammatory erythema with scattered active erosions over abdomen. PDAI: Activity 7, Damage 6. *PDAI*, Pemphigus Disease Area Index.

**Fig 3:**
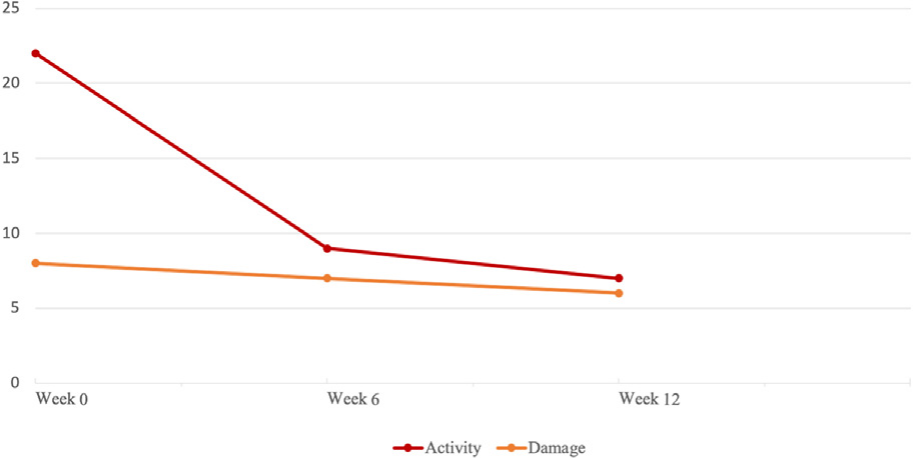
Change in PDAI after commencement of adalimumab. *PDAI*, Pemphigus Disease Area Index.

The role of TNF-α in HHD is not fully elucidated however multiple mechanisms have been suggested. Excessive TNF-α is linked to chronic inflammation and TNF-α modulates inflammation through cytosolic calcium homeostasis, which is impaired in HHD.^9^ TNF-α and IL-6 have been demonstrated to enhance growth of herpes simplex virus 1 in differentiated keratinocytes, exacerbating HHD flares precipitated by viral infections.^3^ IL-6 has also been found to suppress mRNA levels of ATP2C1.^3^ As TNF-α induces IL-6 through the nuclear factor kappa-light-chain-enhancer of activated B cells pathway, TNF-α suppression and subsequent reduction in IL-6 may reduce flares of HHD through promoting ATP2C1 transcription and reducing the effect of the haploinsufficiency that causes the condition.^10^

Adalimumab demonstrated efficacy in this case of HHD both subjectively and when using the PDAI scoring tool. Where first- and second-line agents have failed to adequately control HHD and/or caused side effects, adalimumab may be a promising treatment option and should be considered as a potential third-line agent. This case report reiterates the need for further research into treatment options for this rare but debilitating genodermatosis and highlights anti–TNF-α monoclonal antibodies as a promising avenue for further research.

## Conflicts of interest

Prof Murrell is a cocreator of the Pemphigus Disease Area Index but the license is held by Penn University. Drs Stark and Sheriff have no conflicts of interest to declare.
